# CO_2_ Acts as a Signalling Molecule in Populations of the Fungal Pathogen *Candida albicans*

**DOI:** 10.1371/journal.ppat.1001193

**Published:** 2010-11-18

**Authors:** Rebecca A. Hall, Luisa De Sordi, Donna M. MacCallum, Hüsnü Topal, Rebecca Eaton, James W. Bloor, Gary K. Robinson, Lonny R. Levin, Jochen Buck, Yue Wang, Neil A. R. Gow, Clemens Steegborn, Fritz A. Mühlschlegel

**Affiliations:** 1 School of Biosciences, University of Kent, Canterbury, Kent, United Kingdom; 2 School of Medical Sciences, Institute of Medical Sciences, University of Aberdeen, Aberdeen, United Kingdom; 3 Department of Physiological Chemistry, Ruhr-University Bochum, Bochum, Germany; 4 Department of Pharmacology, Weill Medical College of Cornell University, New York, New York, United States of America; 5 Institute of Molecular and Cell Biology, Agency for Science, Technology and Research, Proteos, Singapore; 6 Department of Biochemistry, University of Bayreuth, Bayreuth, Germany; 7 East Kent Hospitals University NHS Foundation Trust, Clinical Microbiology Service, William Harvey Hospital, Ashford, Kent, United Kingdom; Carnegie Mellon University, United States of America

## Abstract

When colonising host-niches or non-animated medical devices, individual cells of the fungal pathogen *Candida albicans* expand into significant biomasses. Here we show that within such biomasses, fungal metabolically generated CO_2_ acts as a communication molecule promoting the switch from yeast to filamentous growth essential for *C. albicans* pathology. We find that CO_2_-mediated intra-colony signalling involves the adenylyl cyclase protein (Cyr1p), a multi-sensor recently found to coordinate fungal responses to serum and bacterial peptidoglycan. We further identify Lys 1373 as essential for CO_2_/bicarbonate regulation of Cyr1p. Disruption of the CO_2_/bicarbonate receptor-site interferes selectively with *C. albicans* filamentation within fungal biomasses. Comparisons between the *Drosophila melanogaster* infection model and the mouse model of disseminated candidiasis, suggest that metabolic CO_2_ sensing may be important for initial colonisation and epithelial invasion. Our results reveal the existence of a gaseous *Candida* signalling pathway and its molecular mechanism and provide insights into an evolutionary conserved CO_2_-signalling system.

## Author Summary

Pathogenic microorganisms can produce a variety of secondary metabolites and signalling molecules which can affect the host, or provide them with a selective advantage against competing commensal organisms. We demonstrate that gaseous, metabolically generated CO_2_ can serve as a signalling molecule to enhance the organism's virulence during infection establishment by using the fungal pathogen *Candida albicans* as a model. Furthermore, we identified a CO_2_ receptor site within the catalytic domain of the soluble adenylyl cyclase, Cyr1p, which is critical for CO_2_ sensing and hence virulence of the organism. CO_2_ sensing is conserved in a variety of pathogenic species, and increased levels have been shown to suppress the host's immune system. Thus, CO_2_ sensing may represent a mechanism to enhance *C. albicans* virulence when the host's immune system is suppressed.

## Introduction

*Candida albicans* is the predominant fungal pathogen of humans. In healthy individuals *C. albicans* resides as a commensal of the gastrointestinal, oral and vaginal tracts. *C. albicans* can cause superficial infections which, although not life threatening, provide discomfort to the individual and require treatment with antifungals which is a constant drain on hospitals resources. However, *C. albicans* infections are life threatening when the individual's immune system becomes compromised as a result of age, cancer, chemotherapy hospitalisation and AIDS. Under these circumstances superficial infections may readily develop into systemic disease where mortality rates are reported to be up to 40%, which is higher than those for most bacterial infections [1], [2], [3]. For example, oropharyngeal candidiasis is common in patients with haematological malignancies (up to 60%) and those undergoing radiotherapy [4], [5], [6]. Here, a few fungal cells develop into biomasses measuring several millimetres in diameter that penetrate and invade the underlying tissue, eventually leading to dissemination of *Candida* into the blood stream and subsequently systemic infection [7].

Development from superficial infection to invasive disease is mediated by many well characterised virulence factors including morphological transition. *C. albicans* can exist in yeast, pseudohyphal and true hyphal growth forms, all of which are important for the virulence of the organism [8]. Yeast cells are thought to be essential for growth and dissemination [9], while the hyphal forms are essential for invading mucosal membranes [9]. This morphological transition is mediated by host environmental cues including temperature, pH, serum, O_2_, and CO_2_, which the pathogen encounters during disease progression [5], [10], [11].

The virulence-associated morphological transitions of *C. albicans* are largely controlled through the secondary messenger cAMP. In *C. albicans*, cAMP is synthesised by the fungal adenylyl cyclase (AC), Cyr1p [12], a member of the Class III nucleotidyl cyclase family [13]. Activity of Cyr1p governs most processes essential to *C. albicans* virulence including tissue adhesion followed by the invasion of the underlying host-barriers, and biofilm formation [14]. *C. albicans* AC activity is subject to both positive and negative regulation, with an increasing number of molecules directly interacting with specific domains of the protein [10], [15], [16]. For example, bacterial peptidoglycan stimulates Cyr1p via the enzyme's leucine-rich-region [16], and CO_2_/HCO_3_^−^ directly activates the Cyr1p C-terminal catalytic domain [10]. These forms of regulation enable *C. albicans* to recognise and respond (via filamentation) to specific host environmental conditions during disease progression.

In addition to host environmental cues, the morphological transition of *C. albicans* is also regulated by soluble chemical mediators, termed quorum sensing molecules (QSMs). QSMs are secreted into the environment by a variety of microorganisms [for recent reviews see 17], [18], and upon reaching threshold concentrations, impact on microbial behaviour by influencing expression of virulence determinants [19]. QSMs including the self-generated sesquiterpene farnesol [20] and 3-oxo-C12 homoserine lactone (HSL) secreted by *Pseudomonas aeruginosa* [21] inhibit *C. albicans* filamentation though cAMP dependent signalling cascades [22].

Further to soluble chemical mediators, volatile compounds can also act as signalling molecules. For example, in *Saccharomyces cerevisiae*, nutrient limited yeast cells release volatile ammonia, which when sensed by another colony inhibits its growth in the direction of the signal [23]. CO_2_ is a volatile gas that has recently been described as a predominant regulator of *C. albicans* virulence factors and has been shown to effect the virulence of other microbial species [24], [25]. In *C. albicans* CO_2_ functions in two processes key to pathogenicity, one metabolic and the other cell signalling to promote filamentation [10]. In biological systems CO_2_ is maintained in equilibrium with its hydrated form, HCO_3_^−^, via the actions of carbonic anhydrase. HCO_3_^−^ is required for metabolism, but when at high concentrations HCO_3_^−^ directly activates adenylyl cyclase increasing cytosolic cAMP and promoting filamentation [10]. To date, only the effects of high (5%) exogenous CO_2_ concentrations have been investigated in microbial species. However, microbes continuously secrete metabolically generated CO_2_ into their immediate microenvironment at levels perceived to be lower than 5%. Here, we investigate the effects of self generated CO_2_ on pathogenicity associated traits of *C. albicans*. Previously we identified the carbonic anhydrase, Nce103p, as being essential for growth under CO_2_ limiting conditions [10]. Now we explore a new application of the mutant strain Δ*nce103* as a CO_2_ biosensor to report on CO_2_ concentrations within fungal biomasses. Using our CO_2_-dependent bio-sensing strain, we demonstrate that build-up of self-generated, metabolic CO_2_ occurs in a fungal population. Furthermore, we show that CO_2_ mediates its effects as a hierarchy, with low concentrations of CO_2_ functioning to fill metabolic demand, then once CO_2_ exceeds a critical threshold, it promotes filamentation and subsequent surface invasion of the pathogen. We show that microbial CO_2_, like environmental CO_2_, is sensed by the AC catalytic domain and identify a bicarbonate receptor site in Cyr1p.

## Results

### *C. albicans*-generated CO_2_ accumulates under diffusion-limiting conditions

**Figure 1:**
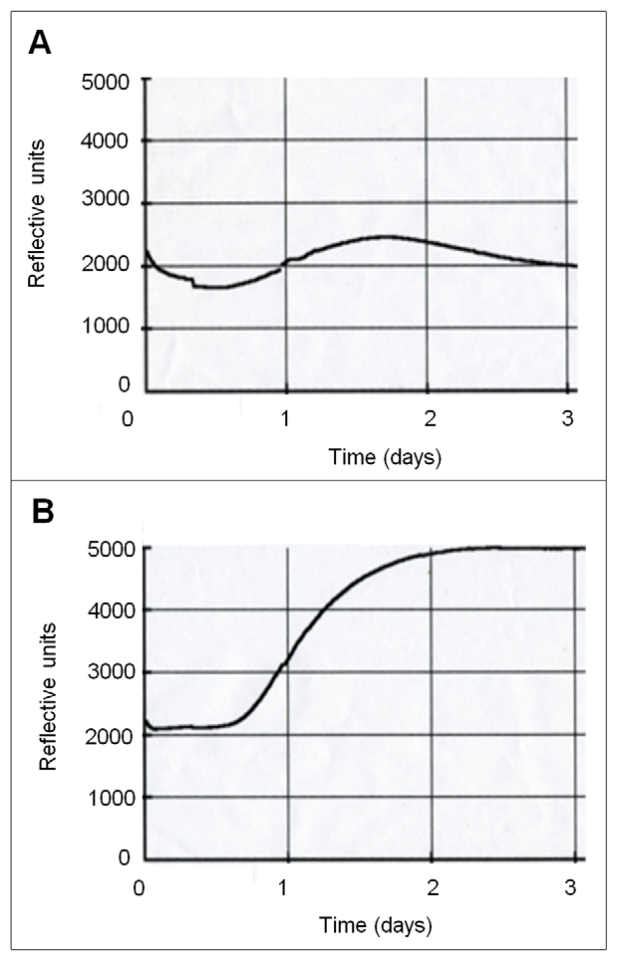
Closed systems enable CO_2_ accumulation. 10,000 Wild type cells were inoculated onto 10 ml DMEM pH7 agar in BacT/ALERT bottles. Bottles were either incubated as an open system (**A**) where free diffusion of metabolically generated gases was permitted or as a closed system (**B**) where diffusion was inhibited. CO_2_ accumulation was measured for 48 hr at 37°C in BacT/ALERT 3D automated microbial detection system (bioMerieux). Reflective units depicted on the X-axis are a direct measurement of the CO_2_ concentration in the system (see material and methods for details).

**Figure 2:**
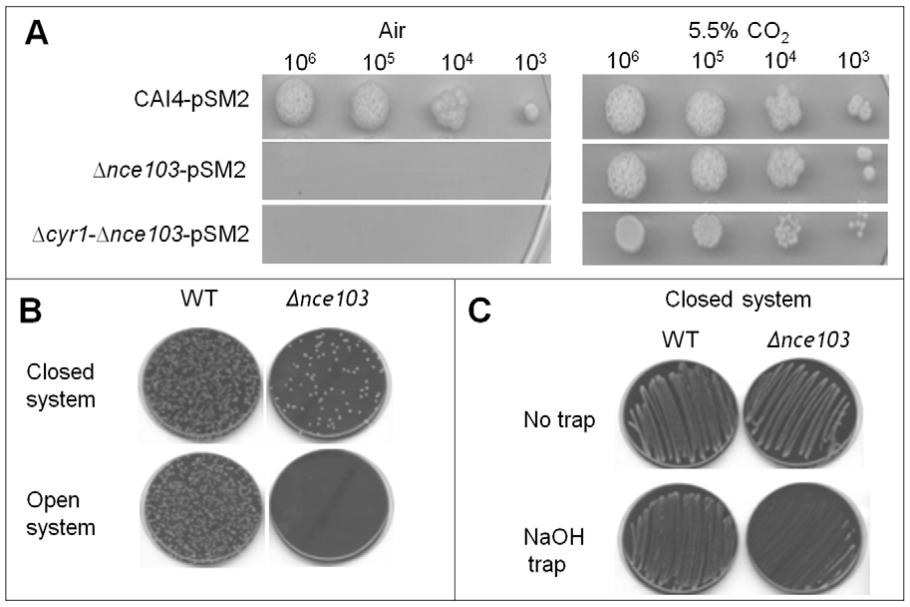
CO_2_ as a self generated volatile communication molecule. **A**) Cell dilutions (5 µl) of CAI4-pSM2, *Δnce103*-pSM2 and Δ*cyr1-Δnce103*-pSM2 were spotted onto YNB plates and incubated in the presence of 0.03% (air) or 5% CO_2_ for 48 hours. **B**) 1000 wild type cells and 200 *Δnce103* cells were inoculated onto separate CBA plates and both plates placed together into zip-locked polyvinyl chloride bags, which were sealed (closed system; top) or left open (open system; bottom) and incubated at 37°C for 48 hours. **C**) Wild type and *Δnce103* cells were incubated in a closed system, as described in B), in the absence or presence of a NaOH trap.

CO_2_ is generated during metabolism and acts as an important cellular signalling molecule in many organisms. CO_2_ influences microbial virulence and organisms behaviours such as mating, feeding or ventilation [26]. We confirmed that, when grown under diffusion-limiting conditions (i.e., closed systems), *C. albicans* accumulated self-generated CO_2_ (Figure 1 A, B). Next we asked whether self-generated CO_2_ could be utilized by *C. albicans* to meet the organism's growth requirements. In normal atmospheres, (0.03% CO_2_) the *C. albicans* carbonic anhydrase (CA), Nce103p, is essential for catalyzing the hydration of CO_2_ to bicarbonate to meet metabolic demands. Therefore, in ‘open’ systems (i.e., under the 0.03% CO_2_ in air), deletion of *NCE103* results in a depletion of bicarbonate levels which inhibits growth. However, at elevated CO_2_ concentrations (such as 5% CO_2_ experienced by *C. albicans* when inside an infected mammalian host) there is sufficient CO_2_ spontaneously hydrated to bicarbonate to meet the metabolic requirements restoring growth (Figure 2A). Therefore, the carbonic anhydrase mutant (TK1; *Δnce103*) can only grow in environments with elevated concentrations of CO_2_ [10], and as a result, functions as a CO_2_ bio-indicator. The *Δnce103* bio-indicator strain failed to grow when co-incubated with wild type (SC5314; WT) cells in an open system, but grew in the presence of WT *C. albicans* in a closed system without exogenously supplied CO_2_ (Figure 2B). Furthermore, incubation of surplus (10,000 CFUs/plate) *Δnce103*, on its own, in closed but not open systems also restored the growth of *Δnce103* (Figure S1), suggesting that in closed systems the elevated CO_2_ levels are sufficient to complement the growth of *Δnce103*.

To confirm that it was volatile CO_2_ generated by the WT strain which restored growth to the *Δnce103* CO_2_ bio-indicator strain, we included hydroxide into the closed system, which specifically traps CO_2_ in the form of carbonate [27]. Solid sodium hydroxide interfered with the growth of the *Δnce103* CO_2_ bio-indicator strain, but not WT (Figure 2C). The diminished growth in the presence of sodium hydroxide is most likely caused by CO_2_ trapping and not oxygen depletion, as oxygen levels are not influenced by the CO_2_ trap. Taken together, these results reveal that metabolically generated CO_2_ can provide sufficient HCO_3_^−^ to meet the metabolic demands of *C. albicans*, and that this CO_2_ can be provided in the form of a volatile signal from neighbouring colonies.

Consistent with the idea that the CO_2_ generated by WT *C. albicans* is supplying CO_2_/HCO_3_^−^ to meet the metabolic demand of the *Δnce103* bio-indicator strain, rescue was independent from the cAMP signalling system, as the *Δcyr1-Δnce103* strain (RH12) was also complemented when incubated at elevated CO_2_ (Figure 2A). These data suggest that there is sufficient CO_2_ generated during normal metabolism of WT *C. albicans* to support the growth of the *Δnce103* bio-indicator strain, as long as diffusion of the generated CO_2_ is limited.

### CO_2_ accumulates inside a fungal biomass

**Figure 3:**
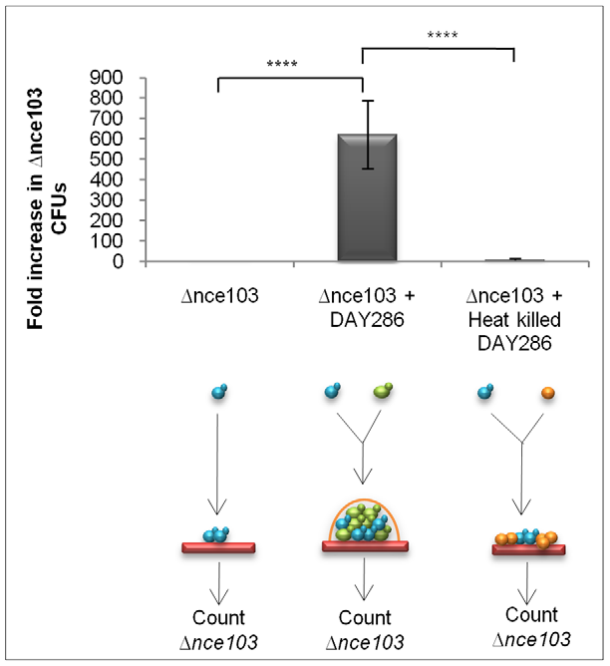
CO_2_ signal build-up occurs within fungal populations. Equal cell numbers (500 CFUs) of DAY286 (represented as green cells in the diagram) and *Δnce103* (represented as blue cells in the diagram), 1000 *Δnce103* alone and 1000 *Δnce103* with 1×10^6^ heat-killed DAY286 (represented as orange cells in the diagram) were spotted onto YPD agar and incubated for 48 hr at 37°C. Cells were recovered, populations separated by plating onto selective media and *Δnce103* CFUs counted. Values are the mean and standard deviation from 8 independent experiments (**** indicates that the P statistic for the represented data was greater than 0.0001).

We next asked whether CO_2_ levels sufficient for signalling would build-up within a fungal biomass. To address this question, we grew the *Δnce103* CO_2_ bio-indicator strain on its own, or mixed with equal numbers of DAY286, a *Δhis1* strain which is wild type for carbonic anhydrase and adenylyl cyclase, in an open system to specifically test whether CO_2_ accumulation could occur between cells growing in the same biomass. Using the different auxotrophic tags (HIS^+^ and HIS^−^) to distinguish the two strains after incubation within mixed biomasses, we were able to directly test whether metabolically generated CO_2_ from DAY286 could complement the growth of *Δnce103*, while *Δnce103* on its own would be restricted in growth. Co-incubation of *Δnce103* with DAY286 enhanced the recovery of *Δnce103* 600-fold (p = >0.0001) when compared to incubation of the CO_2_ bio-indicator strain on its own (Figure 3). To exclude that DAY286 was able to fill the metabolic demands of *Δnce103* by providing other metabolic intermediates other than CO_2_, the *Δnce103* strain was also co-incubated with a surplus (1×10^6^ cells) of heat-killed DAY286 cells. However, co-incubation of *Δnce103* and heat-killed DAY286 did not enhance the recovery of *Δnce103* compared to incubation of the CO_2_ bio-indicator strain alone (Figure 3, p = >0.0001), suggesting that within a fungal biomass, even in an open system, there is an accumulation of metabolic CO_2_ sufficient to promote the growth of *Δnce103*. These data also prove that the carbonic anhydrase is essential because it ‘captures’ metabolically generated CO_2_ as HCO_3_^−^ which is needed to meet metabolic requirements of cells deep within the colony.

### The volatile messenger CO_2_ affects *C. albicans* colony morphology

**Figure 4:**
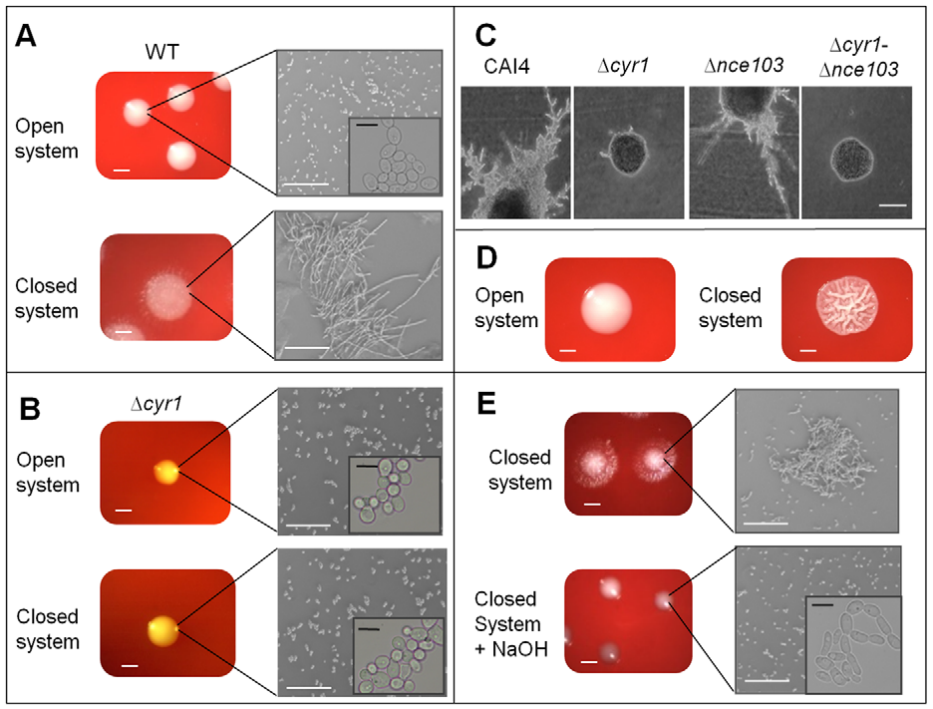
CO_2_ affects *C. albicans* colony morphology. **A**) 500 CFU of wild type SC5314 were seeded onto CBA and incubated under closed and open conditions at 37°C for 48 hr (cells were resuspended at the required cell density, i.e. 5×10^3^ cells/ml, and then 100µl spread plated onto the plates to obtain single colonies (i.e. 500 colonies/plate; scale bar represents 1 mm). Cells were then resuspended in water and viewed at 200× magnification (scale bar represents 100 µm). Insert shows yeast cells at 2000× magnification (scale bar represents 10 µm). **B**) 500 CFU of CR276-CTRL (*Δcyr1*) were seeded onto CBA and incubated in both closed and open systems at 37°C for 72 hr (scale bar represents 1 mm). Cells were then resuspended in water and viewed at 200× magnification (scale bar represents 100 µm). Insert shows yeast cells at 2000× magnification (scale bar represents 10 µm). **C**) CAI4-pSM2, Δ*cyr1-Δnce103*-pSM2 and *Δnce103*-pSM2 cells were streaked onto DMEM pH7 media and incubated at 37°C for 24 hours supplemented with 5% CO_2_. Images were taken at 100× magnification (scale bar represents 100 µm). **D**) SC5314 cells were suspended in sterile water and seeded onto CBA at an initial cell concentration of 50 CFUs and incubated in both closed and open conditions for 48 hrs 37°C (scale bar represents 1 mm). **E**) 500 WT (SC5314) CFUs were incubated on CBA for 48 hr in closed systems either with or without 0.5g of solid NaOH (scale bar represents 1 mm). Single colonies were resuspended in sterile water and images taken at 200× magnification (scale bar represents 100 µm). Insert shows yeast cells at 2000× magnification (scale bar represents 10 µm).

CO_2_ is not only required for metabolism, but it also acts as a signal for cAMP-dependent filamentation of *C. albicans*. Therefore, we sought to determine whether metabolically generated CO_2_ could act within a biomass to modulate morphology. To test whether *C. albicans* produces sufficient CO_2_ to affect filamentation, we incubated wild type cells under open and closed conditions. In open systems, no colonies filamented, while under closed conditions, we observed changes in colony morphology after 48 hours. Plating 500 CFUs produced extensive filamentous colonies (Figure 4A). Microscopic analysis of resuspended colonies confirmed that the majority of the cells filamented in the closed system (forming a highly interwoven mass of cells resistant to mechanical stress; Figure 4A). As *C. albicans* hyphal induction is critically dependent on cAMP signalling cascades [12], we tested a strain deficient for adenylyl cyclase CR276-CTRL (RH20; *Δcyr1*) in our open and closed systems. The *Δcyr1* strain did not show any changes in colony morphology, even when incubation periods were extended to 72 hours to account for its known reduced growth rate (Figure 4B). Additionally, altered morphology was independent of carbonic anhydrase, as *Δnce103*, but not *Δcyr1-Δnce103*, formed filaments in the presence of 5% CO_2_ (Figure 4C). Interestingly, the extent of filamentation of the WT strain was biomass dependent. At 48 hours, plating in the presence of 50 CFUs generated wrinkled colonies (Figure 4D), which were not fully filamentous.

Addition of the hydroxide trap into the closed system inhibited the morphological transition observed previously (Figure 4E). Furthermore, microscopic inspection of WT colonies, after co-incubation with *Δnce103* in closed environments, confirmed that the colonies were smooth, round, yeast colonies, similar to those observed in the open system (data not shown). These observations suggest that the *Δnce103* strain acts as a CO_2_ sink removing the majority of the gas from the system. Therefore, *C. albicans* produces CO_2_ which affects morphology, and cAMP is essential for the observed morphological effects.

### Fungal CO_2_/HCO_3_^−^ sensing is mediated by lysine 1373 of the Cyr1p catalytic domain

Directly testing the *in vivo* relevance of CO_2_ chemosensing would be greatly facilitated by an adenylyl cyclase variant with specifically diminished CO_2_ sensitivity. Previously we have shown that CO_2_/HCO_3_^−^ activates the catalytic domain of the fungal adenylyl cyclase, Cyr1p [10], confirming that this Class III AC belongs to the bicarbonate-responsive soluble AC (sAC) subfamily [13], [28]. Structural studies and *in vitro* work on mutated bacterial sAC-family enzymes indicated a mechanism for bicarbonate regulation, along with a potential bicarbonate binding site [29], [30]. However, it remains to be shown whether the mechanism of activation and potential binding site generally apply to sAC-like enzymes, in particular from eukaryotes, and whether they are responsible for the *in vivo* effects of CO_2_ on AC activity.

**Figure 5:**
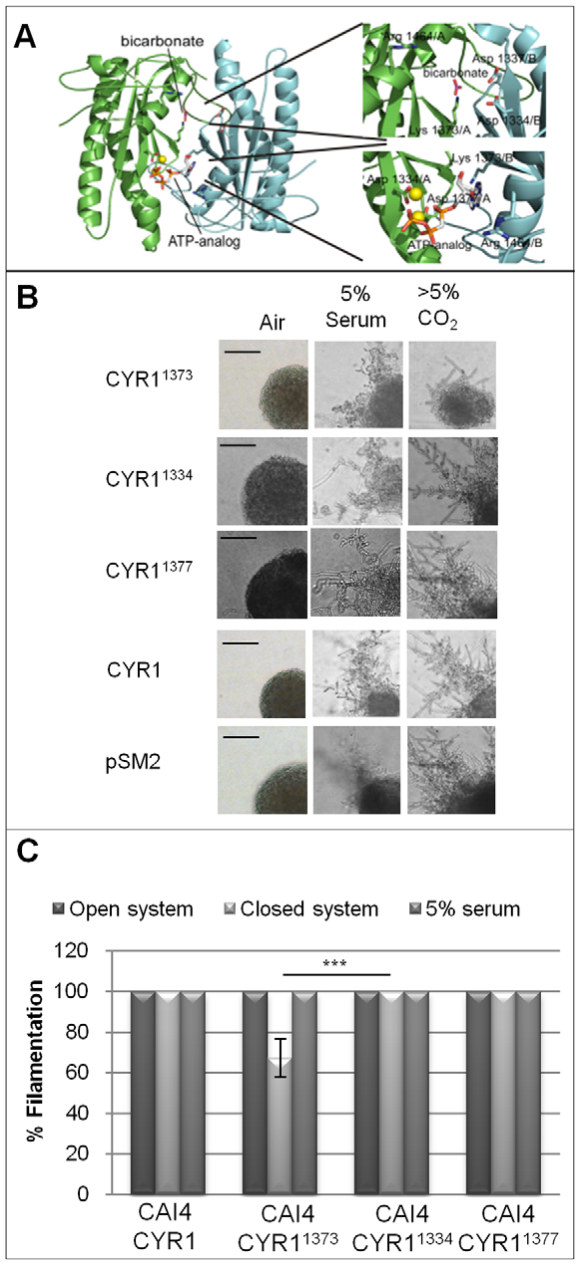
Mechanism of CO_2_ sensing in *C. albicans*. **A**) Homology model of the Cyr1p homodimer with bound substrate analogue and the activator bicarbonate. The protein chains are shown in green and cyan, respectively, and ligands and functional residues mutated in our study are shown in stick representation and labelled with residue number and chain A or B. Lys1373 can either bind the substrate base (active site) or bicarbonate (regulatory site). **B**) Point-mutated *CYR1* (1373, 1334 and 1377 aa), and control plasmids (wt *CYR1* and the vector control pSM2) were integrated into in *URA3* locus of CAI4. Colonies were grown on DMEM pH7 itself, or supplemented with either 5% serum, or 5% CO_2_. The colony depicted for CAI4-CYR1^1373^ displaying an attenuated response to CO_2_ is representative of approximately 30% of the population. The differential response to CO_2_ is hypothesised to result from the variations in levels of CO_2_-responsive Cyr1p homodimers, Cyr1-Cyr1^1373^ heterodimers and non-functional Cyr1p^1373^ homodimers. All other colonies are representative of the entire population (scale bar represents 100 µm). **C**) Cell suspensions of the desired strains were inoculated onto DMEM pH7 supplemented with 10% serum or CBA agar (CBA plates were either incubated in open or closed systems) and after 48 hours colony morphology assessed. Results are the mean and standard deviations from 4 replicate experiments (*** indicates that the P statistic for the represented data is greater than 0.001).

Using sequence alignments of Class III ACs, we generated a homology model of Cyr1p and identified the Cyr1p site corresponding to the proposed bacterial CO_2_ receptor site (Figure 5A) [13], [29]. A lysine residue [29], Lys1373 in *C. albicans* Cyr1p, would be a key interaction partner for bicarbonate in this receptor site. Class III ACs are dimers with shared active sites – i.e. residues from both monomers contribute to each active site – so that only the dimer can display activity. In contrast to ‘heterodimeric’ Class III ACs, which have one active site and a second, related-but-degenerated, ‘regulatory’ site in their dimer interface, homodimeric Class III ACs, like Cyr1p, have two identical catalytic sites in their dimer interface. In these ACs, it is believed that both sites can act as active or as regulatory sites. The putative bicarbonate-interacting lysine residue is strictly conserved in both “active’ and ‘regulatory’ sites (for example, in mammalian sAC, Lys334, would be the corresponding residue in the active site). In active sites, the conserved lysine at this position is essential for substrate binding [13]. Because Lys1373 of Cyr1p should be essential for substrate binding in at least one of the two sites formed at the homodimer interface, we predicted *CYR1^1373^* would be inactive on its own. We integrated full-length Cyr1p with Lys1373 point mutated to alanine, under the control of the *TEF2* promoter, into an adenylyl cyclase null, generating strain CR276-CYR1^1373^ (RH22; *cyr1/cyr1:pTEF2* CYR1^1373^). CR276-CYR1^1373^ was refractory to both CO_2_ and serum induction of filamentation, behaving similarly to the vector-control strain CR276-CTRL (RH20; Figure S2). Thus, *CYR1^1373^* homodimers encode a non-functional AC.

To specifically test the role of this lysine in the bicarbonate activation of Cyr1p and to generate an AC with a selective defect in its bicarbonate responsiveness, we generated strains containing mutant/WT heterodimers. Due to the dimeric architecture of Class III ACs, one wild type Cyr1p monomer could interact with one Cyr1p^1373^ monomer, allowing basal AC activity, but preventing bicarbonate stimulation due to disruption of the bicarbonate interacting site in the second ‘regulatory’ centre (as described above). The point mutated Cyr1p was integrated into a strain expressing wild type adenylyl cyclase, generating strain CAI4-CYR1^1373^ (RH25; *CYR/CYR1/pTEF2 CYR1^1373^*). Consistent with the expected heterodimer formation and with specific interruption of CO_2_-induced cAMP formation, CAI4-CYR1^1373^, but not the control strain CAI4-CYR1 (RH24; *CYR1/CYR1/pTEF2 CYR1*), displayed a signal-specific defect to the filamentation inducing cues (Figure 5B, C), despite the two strains expressing comparable levels of *CYR1*, (Figure S3). CAI4-CYR1^1373^ filamented in response to serum, but much less in response to CO_2_, while the control strain expressing wild type AC, CAI4-CYR1, filamented equally in response to both serum and CO_2_. The incomplete suppression of CO_2_-induced filamentation in CAI4-CYR1^1373^ is consistent with the statistical formation of homodimers and heterodimers between the co-expressed wild type and variant protein, which will also yield fully CO_2_-sensitive wild type homodimers.

To confirm that the observed phenotype is specific to the destruction of the bicarbonate binding site in the *CYR1^1373^* heterodimers, rather than a general influence on AC activity, two additional point mutations, that inactivate Cyr1p stimulus-independent, were constructed. Asp1334 and Asp1377 (involved in active site Mg^2+^ binding) were mutated to Ala and also expressed under the control of the *TEF2* promoter. Integration of these constructs into the adenylyl cyclase mutant (CR272-CYR1^1334^; RH26, and CR276-CYR1^1377^; RH27) confirmed that the proteins were catalytically inactive (Figure S2). However, expression of these inactive proteins in CAI4 (containing two genomic copies of CYR1; CAI4-CYR1^1334^; RH28, and CAI4-CYR1^1377^; RH29) did not perturb hyphal induction in response to 5% serum or elevated CO_2_, as Lys1373 did (Figure 5B, C). Thus, CAI4-CYR1^1373^ shows a specific disruption of CO_2_-induced filamentation and therefore, the identified lysine, which likely acts as bicarbonate binding site, serves as physiological CO_2_ “switch” in Cyr1p and perhaps in all related sAC-type enzymes.

### Self-generated CO_2_ is the volatile signal that accumulates under diffusion-limiting conditions and induces filamentation

We next directly tested whether CO_2_ is the volatile messenger inducing *Candida* filamentation by taking advantage of the CO_2_ insensitive mutant strain CAI4-CYR1^1373^. CAI4-CYR1^1373^ showed an attenuated response in the closed system, with 30% (±9%, P = 0.001) of cells producing smooth colonies indicative of reduced filamentation, while CAI4-CYR1, CAI4-CYR1^1334^ and CAI4-CYR1^1377^ were 100% filamentous (Figure 5C). However, CAI4-CYR1^1373^ cells that were inoculated onto DMEM agar supplemented with 5% serum always produced 100% filamentous colonies, confirming that the reduced filamentation was signal-specific (Figure 5B, C); i.e., CO_2_-induced differentiation was diminished while serum-induced differentiation was unaffected.

### CO_2_ activation of Cyr1p may have a role in pathogenicity

**Figure 6:**
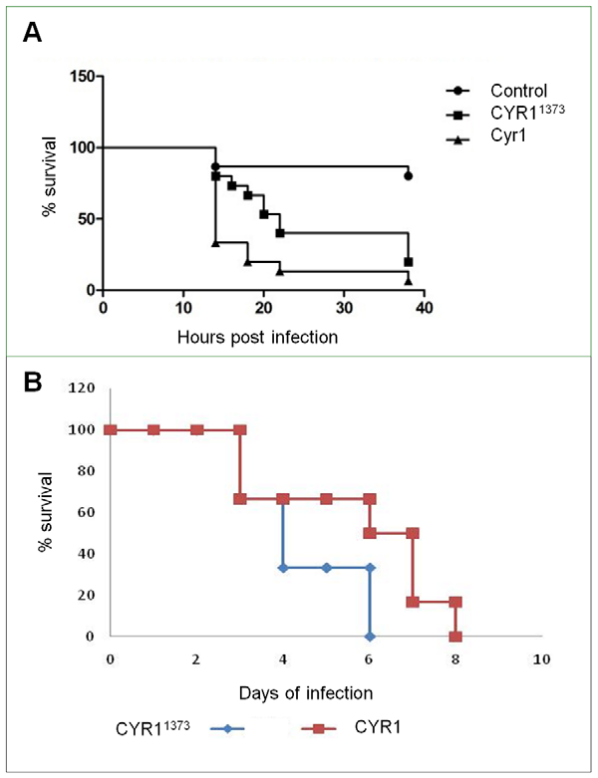
CAI4-CYR1^1373^ may have implications for virulence. **A**) Toll deficient *D. melanogaster* were infected by injection into the thorax with *C. albicans* strains CAI4-CYR1, CAI4-CYR1^1373^ or sterile YDP (CTRL). Experiments were performed with groups of 15 adult files and incubated at 30°C for 38 hours. Values represent the mean and standard deviation from 5 independent experiments. **B**) CAI4-CYR1, CAI4-CYR1^1373^ were intravenously injected (2.4–2.5×10^4^ CFU/g) into 6 female BALB/c mice (6–8 weeks old) and survival monitored. Mice were culled when they showed signs of severe illness or their weight had decreased by more than 20%.

The morphological transition of *C. albicans* is essential to the organism's virulence. As CO_2_ is a potent inducer of hyphal development we tested whether the identified CO_2_-recognition-mechanism regulates *C. albicans* pathogenicity in an *in vivo* model. Initially to test this hypothesis, we selected the Toll-deficient *Drosophila melanogaster* infection model to provide a controlled yet reduced (in respects to filament inducing cues) environment, as only a subpopulation of the cells were CO_2_ insensitive. *D. melanogaster* was infected with CAI4-CYR1^1373^ and CAI4-CYR1 and survival assessed over 48 hours. Although the percentage mortalities of Toll-deficient *D. melanogaster* infected with either strain were similar at the end-point of the time-course experiment, CAI4-CYR1^1373^ killed *D. melanogaster* at a significantly slower rate (p = 0.005) compared to CAI4-CYR1 (Figure 6A). The reduced virulence of CAI4-CYR1^1373^ over CAI4-CYR1 was not attributed to differences in growth rates or fungal burden, as these were comparable between the two strains (Figure S4 and Table S1).

To investigate how the point-mutated adenylyl cyclase would affect the virulence of *C. albicans* in the mammalian host, the mouse model of disseminated candidiasis was utilised. CAI4-CYR1 and CAI4-CYR1^1373^ displayed no significant difference in their ability to cause system infection after intravenous injection (Figure 6B). There was, however, a greater degree of variation in fungal burdens, weight loss and outcome scores compared with control strain (Table S2), which may be reflective of the different populations obtained in the CAI4-CYR1^1373^ strain (i.e. 70% CO_2_ responsive and 30% CO_2_ non responsive).

As the fly model identified that CYR1^1373^ was delayed in its ability to cause infection, we also sampled mice at days 1, 2 and 3 days post-infection to determine whether the delayed ability of CYR1^1373^ to cause infection was also present in the mouse model. However, there were no statistically significant differences in kidney burdens, weight changes or outcome scores, but again there was greater variability in the CAI4-CYR1^1373^ data, which was not observed for the CAI4-CYR1 strain (Table S2). The differences in outcome between the two infection models may be expected. Although the CAI4-CYR1^1373^ strain is reduced in its ability to filament in response to elevated CO_2_ it is responsive to serum or elevated temperature, cues absent in the fly model.

## Discussion

CO_2_ is a biologically important molecule and has major implications for disease progression. As well as host derived CO_2_, microorganisms themselves generate and secrete metabolic CO_2_ into their microenvironment which has the potential to impact on the organism's virulence. We observed that fungal derived, metabolic CO_2_ accumulated in *C. albicans* biomasses to sufficient levels to first provide HCO_3_^−^ as a metabolic intermediate to promote growth and then subsequently to induce the morphological transition crucial for *C. albicans* pathogenicity through activation of Cyr1p via lysine residue 1373.

CO_2_ is produced by multiple metabolic processes and the data presented here suggest that nutrient availability affects production rates. For instance, we found that fungal biomasses grown on nutrient rich media (YPD) were able to support the growth of over ten times the amount of our bio-indicator strain (*Δnce103*) compared to those grown on nutrient limiting media (YNB; data not shown). This result may reflect the increased flux through metabolic pathways as the organism utilises the available nutrients. In accordance with this Ghosh *et al.* recently proposed that the catalysis of arginine to urea and urea's subsequent breakdown to CO_2_ produces sufficient CO_2_ to induce *C. albicans* germ tube formation when engulfed by macrophages [31]. Therefore, arginine biosynthesis maybe a key contributor to CO_2_ production in *C. albicans*.

Accumulation of metabolically generated CO_2_ in race tubes has been shown to impact on asexual spore development in *Neurospora crassa* [32], [33]. Here, simple displacement of the accumulated CO_2_ (by inverting the tubes) restores conidial banding. These results suggest that the heavier density of CO_2_ compared to O_2_ and N_2_ allow it to accumulate in a system more freely rather than diffusing away. In accordance with this, we found that growth of the *Δnce103* strain was enhanced at the bottom of the colony (17-fold, P = 0.001) where agar invasion was observed to stem from the centre of the colony, suggesting that the concentration of CO_2_ is highest at the lower extremities of the biomass (data not shown).

The ability to accumulate in a system is essential for communication molecules, with many molecules only having an impact once a threshold concentration is reached. However, unlike conventional QSMs, CO_2_ may not be specifically generated for the purpose of communication. This is mainly due to the lack of evidence for a single pathway controlling CO_2_ output, although the work of Ghosh *et al* suggest that arginine biosynthesis may play a significant role in the production of CO_2_ in *C. albicans* [31]. Therefore, it is more likely that the organisms have evolved to sense and respond to CO_2_ gradients as a form of diffusion sensing rather than CO_2_ being a true quorum sensing molecule.

However, the interplay between CO_2_ production and other microbial species maybe relevant. When colonising mucosal membranes and epithelia *C. albicans* will be in contact with other microbes residing in the same niche. For example we found that under diffusion limiting conditions significantly fewer colony forming units (10-fold less) of *Escherichia coli* or *Pseudomonas aeruginosa* were required to restore growth of the CO_2_ bio-indicator strain, *Δnce103*, compared to wild type *C. albicans* (data not shown). Given that *C. albicans* is found in mixed microbial biofilms on medical devices it is interesting to speculate about the role the metabolically generated CO_2_ in biofilm establishment and maintenance.

Signalling molecules normally interact with membrane associated receptors to initiate intracellular signalling cascades terminating in a transcriptional response which subsequently induces the desired effect. Unlike most signalling molecules, CO_2_ enters the cell by simple diffusion and is maintained in the cell through hydration to HCO_3_^−^ via the actions of carbonic anhydrase. Although HCO_3_^−^ is a metabolic intermediate and will feed into various metabolic processes, a conserved HCO_3_^−^ binding site was identified in the adenylyl cyclase, Cyr1p, involving lysine residue 1373, which enables CO_2_/HCO_3_^−^ to bind and directly stimulate Cyr1p and hence activate cAMP dependent signalling cascades. Mutation of the HCO_3_^−^ binding site resulted in a subpopulation of cells that were CO_2_ non responsive.

**Figure 7:**
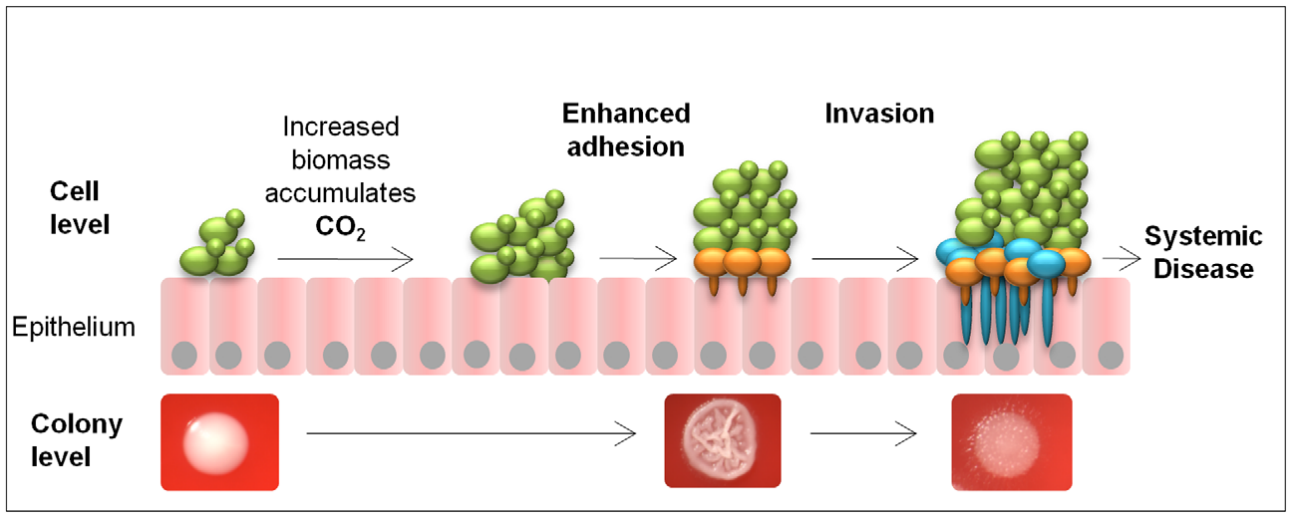
Model for metabolic CO_2_ signalling in fungal pathogenicity. As the cells proliferate on the epithelial surface, increasing fungal growth generates pockets of elevated CO_2_ located at the bottom of the biomass. Cells exposed to the elevated CO_2_ undergo morphological switching, promoting hyphal development and hence increasing the adherence of the organism. At the same time, the protruding hyphae would expose the pathogen to host environmental signals like serum, pH and further increases in CO_2_ levels, which would further enhance hyphal development, increasing the opportunity for tissue invasion.

Introduction of the CO_2_ sensing deficient strain (CAI4-CYR1^1373^) into the Toll-deficient *D. melanogaster* infection model highlighted its reduced ability to kill the host. In the mouse model for disseminated candidiasis this attenuated virulence was not observed. However, this was hypothesised as the mutated strain remained fully responsive to other host environmental cues, including the elevated temperature and presence of serum in mammals, which are absent in the fly infection model. Taking this into consideration we hypothesise that the ability to sense and respond to metabolically generated CO_2_ gradients is important during colonisation and initial invasion of mucosal membranes lining the oral and vaginal tracts during superficial infections where environmental CO_2_ conditions are low and not as important during systemic infection (Figure 7). Here, in an expanding fungal biomass self produced metabolic CO_2_ gradually accumulates and once reaching threshold concentrations directly activates the soluble adenylyl cyclase, Cyr1p via the catalytic, bicarbonate receptor site. The resulting increase in cytosolic cAMP, in conjunction with other epithelial adhesion mechanisms, functions to induce the morphological switch in *C. albicans*. Hyphal formation results in the penetration and invasion of the underlying epithelial cells, which subsequently enhances the dissemination of the fungal pathogen. Our data supports this as we routinely found enhanced levels of *Δnce103* cells in the biomass sections that were invading into the agar, similar to what is observed in oropharyngeal candidiasis, suggesting that cells towards the bottom of the biomass are exposed to higher concentrations of CO_2_ than cells on the surface, which would support hyphal development. Therefore, we hypothesise that during superficial infections that occur in niches where environmental CO_2_ concentrations are low (for example, on the skin and mucosal membranes lining the oral cavity) *C. albicans* can use self generated, metabolic CO_2_ to enhance adhesion and promote filamentation of the underlining cells increasing the opportunity for dissemination into the bloodstream.

In line with CO_2_ playing an enhancing role in microbial virulence, hypercapnia (elevated CO_2_) has recently been shown to inhibit the production of anti-microbial peptides in *Drosophila* [34]. Furthermore, elevated CO_2_ levels suppress the mammalian inflammatory response [35], [36], [37]. Therefore, pathogen associated, metabolically generated CO_2_ may play multiple roles in the infection process. One would operate at a local level, suppressing the host's immune system in the underlining epithelia and rendering the host susceptible to infection. Secondly, high CO_2_ would enhance the microbe's pathogenicity, providing more opportunity for host cell invasion.

In conclusion, Cyr1p is a multifunctional sensor that is essential to fungal pathology. It contains multiple domains that mediate signal-specific enzyme activation in *C. albicans* in response to diverse filamentation-inducing molecules. We have now identified the mechanism by which this AC is stimulated *in vitro* and *in vivo* by CO_2_, supplied by the environment or the fungal biomass itself. Our results give novel molecular insights into this pathogenicity mechanism, as well as an evolutionary conserved CO_2_-chemoreception system. Interfering with fungal CO_2_-sensing may reveal novel approaches for therapeutic intervention.

## Methods

### Ethics statement

All animal experimentation was done in accordance with United Kingdom Home Office regulations and was approved by both the Home Office and the University of Aberdeen ethical review committee. All mice were checked and weighed at least once daily, and if they showed any signs of severe disease and/or had lost 20% of their original body weight mice were humanely terminated immediately. Mice sampled at defined time points were also humanely terminated prior to aseptic removal of kidneys for burden determination.

### Strains and media

*C. albicans* strains and transforming plasmids used in this study are listed in Table S3. Columbia blood agar plates (CBA), a quality-controlled growth medium routinely used in diagnostic microbiology laboratories, supplemented with 5% defibrinated horse blood were either purchased premade, or were made from Columbia blood agar base [38] from Oxoid (2.3% peptone, 0.1% starch, 0.5% NaCl, 1% agar, pH 7.3). Dulbecco's Modified Eagle Medium (DMEM) without bicarbonate and pyruvate was obtained from GIBCO and used at pH7, (1.34% DMEM, 3.57% HEPES supplemented to a final concentration of 2% glucose). YNB and YPD were made as described previously [10]. Where supplementation with 5% CO_2_ was required, plates were incubated in a CO_2_ incubator (Infors HT Minitron) enriched with 5% (vol/vol) CO_2_. Solidified or serum supplemented media contained 2% agar and 5% horse serum.

Toll transheterozygotes flies were generated by crossing flies carrying a loss of function allele of Toll (*Tl^1-RXA^*; obtained from the Tübingen Drosophila Stock Collection) and flies carrying a thermo-sensitive allele of Toll, with a strong phenotype at 29°C (*Tl^3^*; obtained from the Bloomington Stock Center). All stocks were maintained on standard fly medium at 25°C, except during infection experiments where flies were incubated at 30°C.

### Open and closed systems

For diffusion-permitting (open) systems, plates were incubated in the standard way with no additional sealing mechanism. To generate a diffusion-limiting (closed) environment, standard 10 cm petri dishes containing CBA (20 ml) were sealed with two layers of laboratory sealing film (Parafilm) followed by three layers of standard cling-film (low density polyvinyl chloride). To minimise diffusion the sealing process was repeated twice. When plates were to be incubated in parallel, standard petri dishes were placed into, zip-locked polyvinyl chloride bags (15.5×23 cm). To ensure that the bags were air tight they were sealed mechanically with an additional polyethylene bar making the bags both air and water tight.

### Measurement of CO_2_ accumulation in diffusion-limiting conditions

Sodium hydroxide was used as a CO_2_-trap as described by the equation below. Plates were incubated in air tight plastic bags containing a separate vial of 4M NaOH, or 0.5g of solid NaOH crystals for 48 hrs. To measure CO_2_ accumulation the BacT/ALERT system [39] was used with some modifications. The prefilled bottles were emptied in a sterile environment, media replaced with 20 ml of solidified DMEM pH7 and the agar surface seeded with 10,000 SC5314 cells. Bottles for incubation in closed systems were sealed as described for agar plates. CO_2_ accumulation was directly measured using a BacT/ALERT 3D automated microbial detection system (bioMerieux) where microbial CO_2_ production is assessed by a colorimetric sensor and detection system (red L.E.D and red-light-absorbing photodiode). Emitted light is recorded as a voltage signal that is directly proportional to the reflective light and hence the concentration of CO_2_ in the bottle.
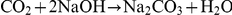



### Heterogeneous populations of CAI4 and *Δnce103*

Heterogeneous cell suspensions containing equal proportions (500 cells/µl) of DAY286 and *Δnce103* were spotted (1 µl total) onto individual YPD or YNB plates and incubated at 37°C for 48 hrs. Initially 1 ml of sterile water was used to wash the single colony from the plate with light agitation of the agar to remove adhered cells. From the recovered 800 µl, 200 µl was plated onto YNB, 5% CO_2_ to promote growth of the strictly CO_2_-requiring strain *Δnce103* strain only (DAY286 will not grow under these conditions as it is *Δhis1/Δhis1*). Stability of the different phenotypic markers was verified upon replica-plating of colonies. The number of colonies was counted, and after taking into account the dilution factor, related back to the initial number of colonies in the cell suspension. Initial cell suspensions were always replica plated onto YNB and YPD to obtain the average starting cell concentration for each strain.

### Molecular modelling of a nucleotide and bicarbonate complex of homodimeric Cyr1p catalytic domain

The amino acid sequence of Cyr1p was duplicated and aligned with the sequences of two chains of a homodimeric substrate analogue complex of CyaC from *Spirulina platensis* (PDB ID 1WC0; [30]) by using Genedoc (http://www.psc.edu/biomed/genedoc). A homology model for Cyr1p was generated with this alignment using Modeller [40], and nucleotide and divalent ions positioned by superposition with the experimentally determined CyaC complex structure. Bicarbonate was then positioned manually at the site proposed for binding in bacterial sAC-like enzymes [13]. The model was visualized using Pymol (DeLano Scientific; http://www.pymol.org).

### Site directed mutagenesis of *CYR1*

Lys 1373, Asp 1334 and Asp 1377 were point mutated to Ala by site directed mutagenesis using the following sets of primers (mutations underlined) 1373F-tggatatgaagtggcgactgaaggtgatg and Primer 7-ctatttaagttcattaactgttttcatgat, Primer 8-aacttgtttcactcccagca and 1373R-atcaccttcagtcgccacttcatatccac, 1334F-ggttttcactgcgatcaaaaactcaac and Primer 7, 1334R-gttgagtttttgatcgcagtgaaaacc and Primer 8, 1377F-gactgaaggtgcggcgttcatgg and Primer 7, 1377R-ccatgaacgccgcaccttcagtc and Primer 8. The resulting PCR fragments were ligated into the SpeI and BamHI restriction sites of pSM2. The 5′ domain of CYR1 together with the *TEF2* promoter were subsequently ligated into the pSM2 plasmid using XbaI and HindIII (site located with C-terminal domain of CYR1) restriction sites forming pACL1, pACL2, and pACL3. Full-length, native CYR1 cloned into pFM2 under the control of the TEF2 promoter was subsequently restricted using SacI and BamHI restriction sites and ligated into pSM2 forming plasmid pSMTC. Plasmids pACL1, pACL2, pACL3, pSMTC and pSM2 (vector control) were integrated into the *URA3* locus of CR276 and CAI4, generating strains RH20-25 (Figure 7A) using standard heat-shock procedures as previously described [41].

### Southern blot analysis

Single copy integration of pACL1 was confirmed for five resulting CAI4 transformants by southern analysis using DIG High primer DNA labelling and detection (Roche) as per the manufacturer's recommendations. DNA probe (1 kb) was PCR amplified using primer-44 5′TTGGTGACATTGAGGCGTTA and primer-47 5′GTTCAATTGTCATTCCGGCAT.

### Semi-quantitative RT-PCR

To assess transcript levels of *CYR1* total RNA was extracted from cultures (50 ml YPD) grown to OD_600_ 0.5. Cells were harvested through centrifugation and immediately frozen in liquid nitrogen. Samples were disrupted using a Mikro-dismembrator S (Sartouis) at 2000 rpm, 2 minutes and RNA immediately extracted using the Qiagen RNeasy Kit according to the manufacturer's recommendations. *CYR1* expression levels (native *CYR1* and *CYR1^1373^*) were analysed by semi quantitative RT-PCR using the BioRad one-step RT-PCR Kit with Syber Green (primers CYR1-F 5′GACGACAACAAACGTGCCAGAACA and CYR1-R 5′ AATCACGTGCTGAAACATGGTCCC). *CYR1* levels were normalised to ACT1.

### Disruption of *NCE103*

The strain, RH12 (*Δnce103 Δcyr1*), was constructed in the *Δcyr1* background strain, CR276, using the *HisG-URA3-HisG* cassette to disrupt the 847 bp *NCE103* open reading frame (GenBank association number EAL03010) from positions +153 to +807 as described previously [10]. Correct integration of the *HisG-URA3-HisG* cassette into the *NCE103* locus was confirmed by PCR.

### Mouse infection models

#### Survival experiments

CAI4-CYR1 and CAI4-CYR1^1373^ were grown on YPD plates at 30°C for 16 hrs and cells were washed off plates with saline, the resulting cell suspensions washed twice with sterile saline and then resuspended in saline to provide inocula for infection. For each *C. albicans* strain 6 female (6–8 weeks old) BALB/c mice (Harlan, UK) were intravenously challenged with 2.4–2.5×10^4^ CFU/g of each strain. Mice were monitored and weighed at least once daily, with mice culled when they displayed signs of severe illness, or when their weight decreased by 20%. When culled, the kidneys were aseptically removed and burdens determined. Survival data were compared by the Kaplan-Meier Log-rank statistic.

#### Outcome scores

For each *C. albicans* strain 9 mice were challenged intravenously, as described above, with three mice sampled on days 1, 2 and 3 post-infection. For each mouse kidney burdens and percentage weight change were determined, with the two parameters used to determine infection outcome scores [42]. Kidney burdens, weight change and outcome scores were compared by the Mann-Whitney U statistic.

## Supporting Information

Figure S1 *Δnce103* can promote its own growth under diffusion limiting conditions. 10,000 CFUs of *Δnce103* were plated onto CBA media and incubated in open or closed systems for 48 hr.
(0.58 MB TIF)

Figure S2 Mutations in *CYR1* affect adenylyl cyclase activity as homo, but not heterodimers (related to Figure 5). The desired point mutated adenylyl cyclase genes or control plasmids were integrated into the *URA3* locus of the adenylyl cyclase mutant (CR276). Resulting transformants were screened on DMEM pH7, DMEM pH7 supplemented with 5% serum and DMEM pH7 incubated in atmospheres of 5.5% CO_2_. Plates were incubated at 37°C for 24 hrs. Scale bar represents 100 µm.
(0.58 MB TIF)

Figure S3 Expression levels of *CYR1* constructs (related to Figure 5 and Figure 6). **A)** Schematic diagram of the *CYR1* locus and the CAI4 *URA3* locus containing the integrated cassette. **B)** Strains were checked for single copy integration of plasmids containing CYR1 and CYR1^1373^. Genomic DNA from the parental strain CAI4 (Lane 1), CAI4-CYR1, strains (Lanes 2 and 3) and CAI4-CYR^11373^ strains (Lanes 4–8) was digested with Hind III and detected using 1Kb probe specific to the 3′ CYR1 open reading frame. **C)** Expression levels of *CYR1* in the parental control strain, CAI4-CYR1 and CAI4-CYR1^1373^ as analysed by semi quantitative RT-PCR. Values are the mean and standard deviation from two independent experiments.
(0.45 MB TIF)

Figure S4 CAI4-CYR1^1373^, CAI4-CYR1 and CAI4-pSM2 have the same growth rates (related to Figure 6). Overnight cultures were diluted to an initial OD_600_ 0.1 in fresh YPD and growth rate followed at 37°C, 150 rpm for 9 hours. Values represent the mean and standard deviation from two independent experiments.
(0.14 MB TIF)

Table S1 Fungal burden in the *D. melanogaster* infection model (related to Figure 6A). Flies were homogenised in sterile water and CFUs determined on YPD agar supplemented with chloramphenicol.
(0.05 MB RTF)

Table S2 Mouse infection parameters measured on day 1–3 post-infection (related to Figure 6B). For each *C. albicans* strain 9 mice were challenged intravenously, with three mice sampled on days 1, 2 and 3 post-infection.
(0.08 MB RTF)

Table S3 Strains used in the study.
(0.12 MB RTF)
