# Supplementary figures and images for: CO2 Acts as a Signalling Molecule in Populations of the Fungal Pathogen Candida albicans
Source: PLoS Pathog. 2010 Nov 18;6(11):e1001193. doi: 10.1371/journal.ppat.1001193 (PMC2987819; doi:10.1371/journal.ppat.1001193)

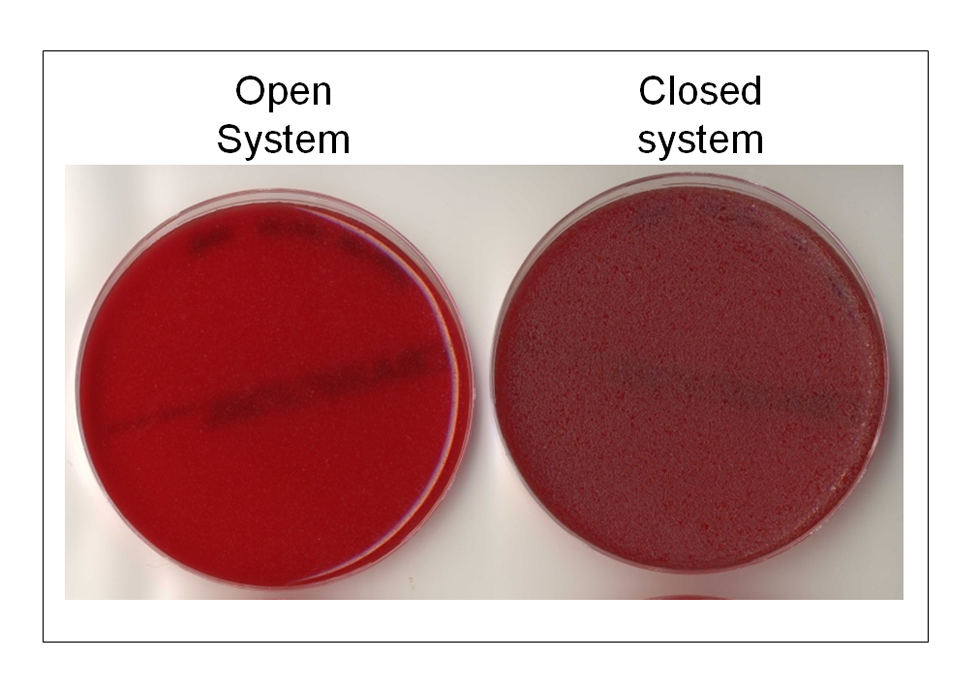

Supplement: Figure S1 — Δnce103 can promote its own growth under diffusion limiting conditions. 10,000 CFUs of Δnce103 were plated onto CBA media and incubated in open or closed systems for 48 hr. (0.58 MB TIF) [file ppat.1001193.s001.tif]

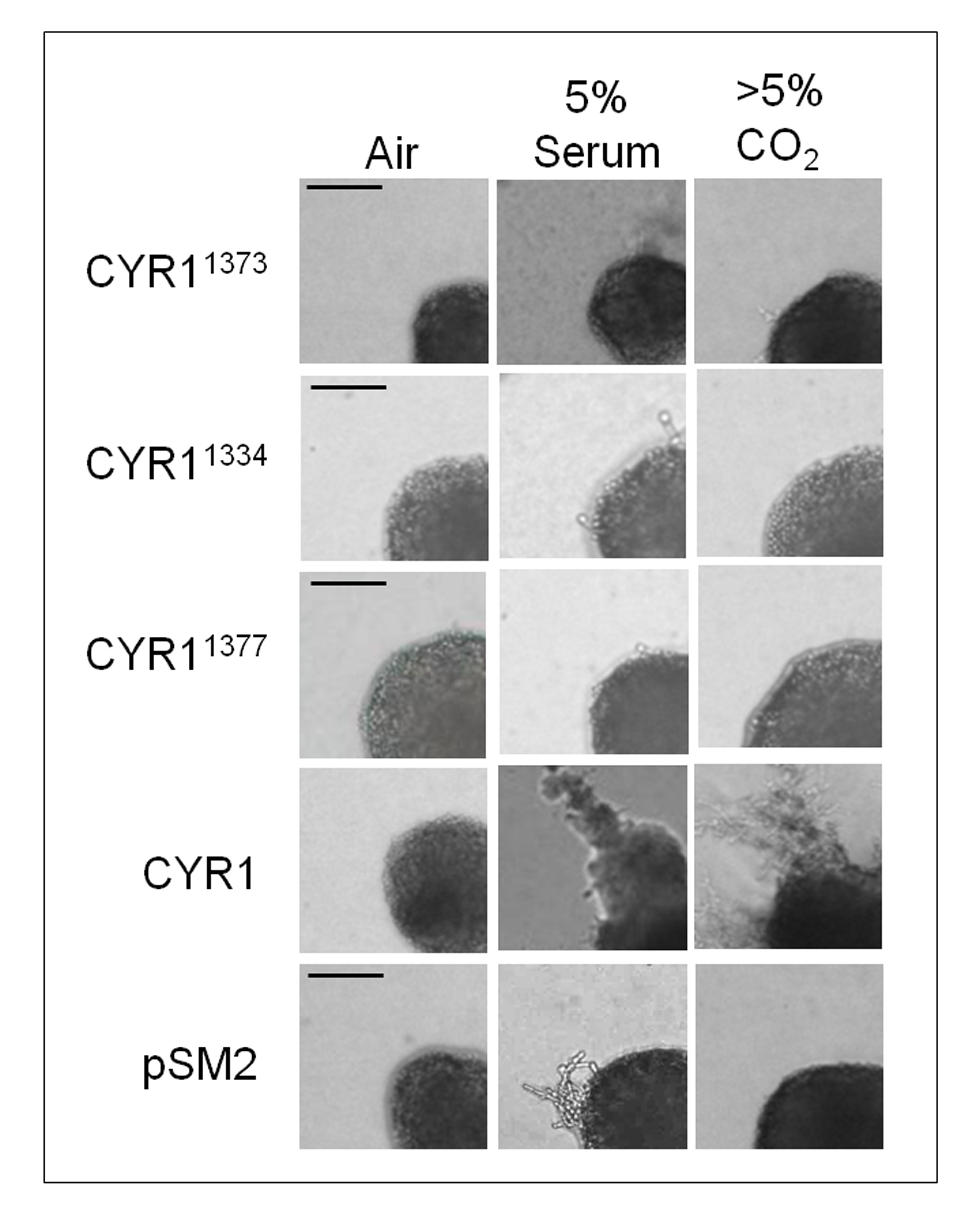

Supplement: Figure S2 — Mutations in CYR1 affect adenylyl cyclase activity as homo, but not heterodimers (related to Figure 5). The desired point mutated adenylyl cyclase genes or control plasmids were integrated into the URA3 locus of the adenylyl cyclase mutant (CR276). Resulting transformants were screened on DMEM pH7, DMEM pH7 supplemented with 5% serum and DMEM pH7 incubated in atmospheres of 5.5% CO2. Plates were incubated at 37°C for 24 hrs. Scale bar represents 100 µm. (0.58 MB TIF) [file ppat.1001193.s002.tif]

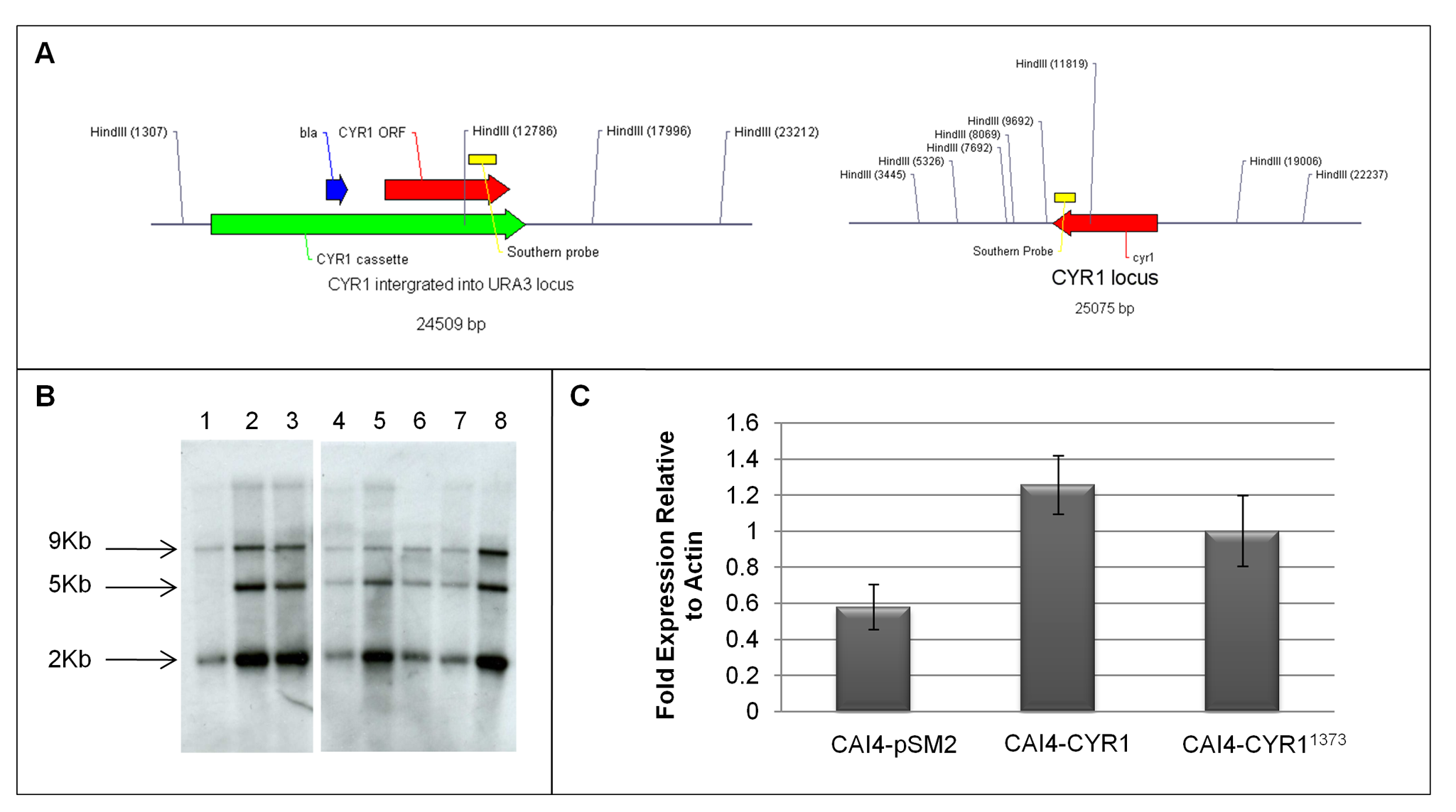

Supplement: Figure S3 — Expression levels of CYR1 constructs (related to Figure 5 and Figure 6). A) Schematic diagram of the CYR1 locus and the CAI4 URA3 locus containing the integrated cassette. B) Strains were checked for single copy integration of plasmids containing CYR1 and CYR11373. Genomic DNA from the parental strain CAI4 (Lane 1), CAI4-CYR1, strains (Lanes 2 and 3) and CAI4-CYR11373 strains (Lanes 4–8) was digested with Hind III and detected using 1Kb probe specific to the 3′ CYR1 open reading frame. C) Expression levels of CYR1 in the parental control strain, CAI4-CYR1 and CAI4-CYR11373 as analysed by semi quantitative RT-PCR. Values are the mean and standard deviation from two independent experiments. (0.45 MB TIF) [file ppat.1001193.s003.tif]

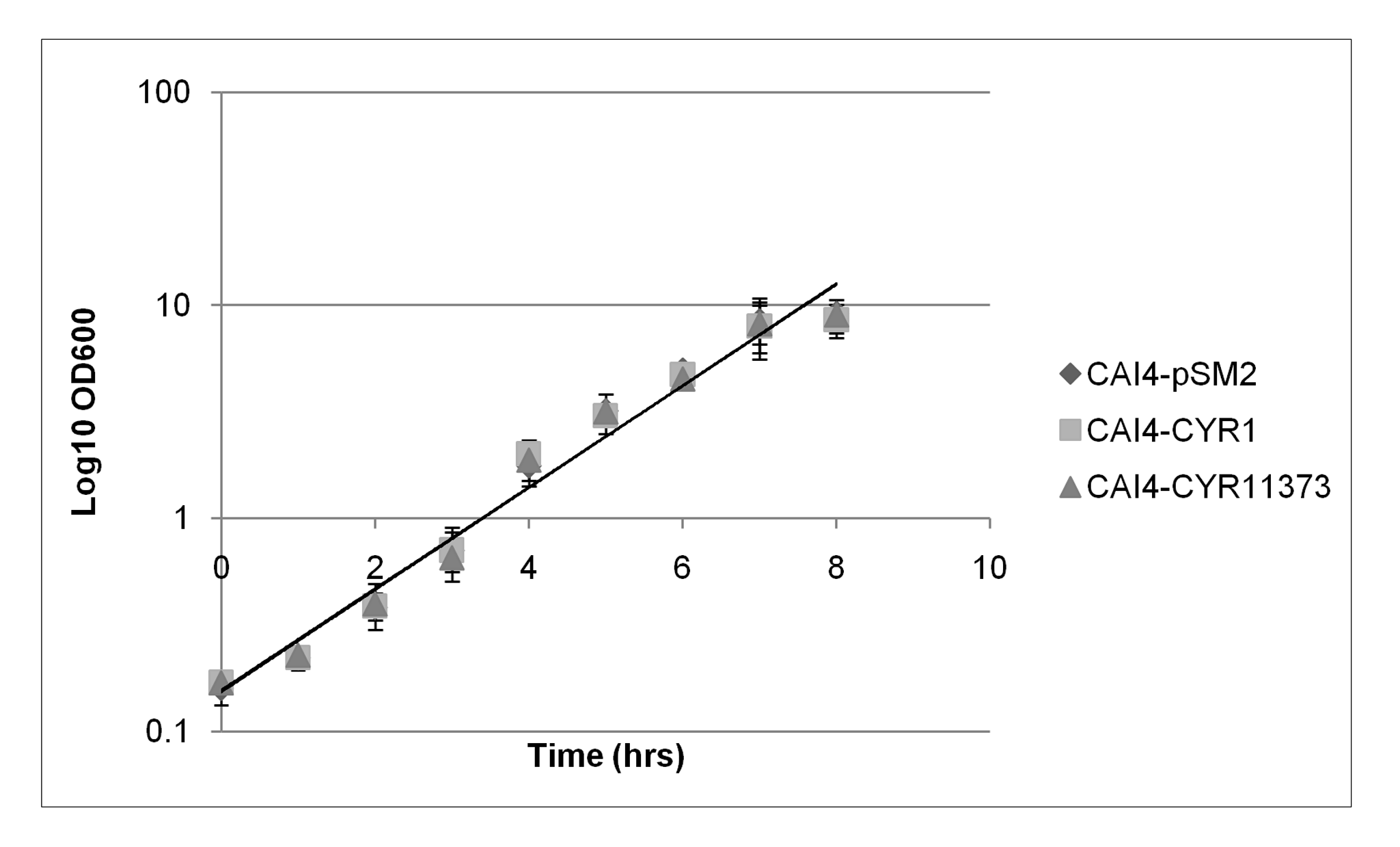

Supplement: Figure S4 — CAI4-CYR11373, CAI4-CYR1 and CAI4-pSM2 have the same growth rates (related to Figure 6). Overnight cultures were diluted to an initial OD600 0.1 in fresh YPD and growth rate followed at 37°C, 150 rpm for 9 hours. Values represent the mean and standard deviation from two independent experiments. (0.14 MB TIF) [file ppat.1001193.s004.tif]
