# Supplementary material for: CO2 Acts as a Signalling Molecule in Populations of the Fungal Pathogen Candida albicans
Source: PLoS Pathog. 2010 Nov 18;6(11):e1001193. doi: 10.1371/journal.ppat.1001193 (PMC2987819; doi:10.1371/journal.ppat.1001193)
Supplement: Table S1 — Fungal burden in the D. melanogaster infection model (related to Figure 6A). Flies were homogenised in sterile water and CFUs determined on YPD agar supplemented with chloramphenicol. (0.05 MB RTF) [file ppat.1001193.s005.rtf]

Table S1. D. melanogaster fungal burdens (Related to Figure 6A) 
Hours	0	16	20	38	
CAI4-CYR1	21666 (± 10408)	120000 (± 65574)	1750000 (± 1890106)	9766667 (± 1201388)	
CAI4-CYR11373	11600 (± 7119)	104000 (± 80672)	1373333 (± 1533504)	8500000 (± 1539480)	
P value	0.0300	0.1039	0.1087	0.02441	
