# Supplementary material for: CO2 Acts as a Signalling Molecule in Populations of the Fungal Pathogen Candida albicans
Source: PLoS Pathog. 2010 Nov 18;6(11):e1001193. doi: 10.1371/journal.ppat.1001193 (PMC2987819; doi:10.1371/journal.ppat.1001193)
Supplement: Table S2 — Mouse infection parameters measured on day 1–3 post-infection (related to Figure 6B). For each C. albicans strain 9 mice were challenged intravenously, with three mice sampled on days 1, 2 and 3 post-infection. (0.08 MB RTF) [file ppat.1001193.s006.rtf]

Table S2. Infection parameters measured on days 1-3 post-infection (Relates to Figure 6B)
	 	Kidney burdens 
[log 10 (CFU/g)]	 	Weight change (%)	 	Outcome Score 	
 		mean	stdev		mean	stdev		mean	stdev	
Day 1									 	
CAI4-CYR11373		6.0	0.3		-6.1	3.6		9.0	2.0	
CAI4-CYR1		5.8	0.2		-7.7	2.8		9.6	1.5	
 									 	
Day 2									 	
CAI4-CYR11373		6.3	1.4		-11.9	5.6		12.2	4.2	
CAI4-CYR1		5.9	0.1		-9.0	1.8		10.4	1.0	
 									 	
Day 3									 	
CAI4-CYR11373		6.3	1.7		-13.8	7.7		13.2	5.5	
CAI4-CYR1	 	6.2	0.4	 	-18.6	1.1	 	15.5	0.3	
