# Supplementary material for: CO2 Acts as a Signalling Molecule in Populations of the Fungal Pathogen Candida albicans
Source: PLoS Pathog. 2010 Nov 18;6(11):e1001193. doi: 10.1371/journal.ppat.1001193 (PMC2987819; doi:10.1371/journal.ppat.1001193)
Supplement: Table S3 — Strains used in the study. (0.12 MB RTF) [file ppat.1001193.s007.rtf]

Supplemental Table 3, C. albicans strains and plasmids used in this study
Name	Genotype	Reference	
SC5314	Wild type	[1]	
CAI4	ura3::l imm434 ura3::l imm434	[2]	
DAY286	ura3::imm434 his1::hisG pARG4::URA3::arg4::hisG, ura3::imm434 his1::hisG arg4::hisG	[3]	
			
			
			
CR276	ura3::l imm434/ura3::1 imm434 cdc35::hisG/cdc35::hisG	[4]	
			
TK1	ura3::l imm434 ura3::l imm434
nce103::HisG/ nce103::HisG (pSM2)	[5]	
RH12	ura3::l imm434 ura3::l imm434 nce103::HisG/ nce103::HisG cyr1::HisG/cyr1::HisG	This study	
RH20	ura3::l imm434/ura3::1 imm434 cyr1::hisG/cyr1::hisG (pSM2)	This study	
RH21	ura3::l imm434/ura3::1 imm434 cyr1::hisG/cyr1::hisG (pSMTC)	This study	
RH22	ura3::l imm434/ura3::1 imm434 cyr1::hisG/cyr1::hisG (pACL-1)	This study	
RH26	ura3::l imm434/ura3::1 imm434 cyr1::hisG/cyr1::hisG (pACL-2)	This study	
RH27	ura3::l imm434/ura3::1 imm434 cyr1::hisG/cyr1::hisG (pACL-3)	This study	
RH23	ura3::l imm434 ura3::l imm434 (pSM2)	This study	
RH24	ura3::l imm434 ura3::l imm434 (pSMTC)	This study	
RH25	ura3::l imm434 ura3::l imm434 (pACL-1)	This study	
RH28	ura3::l imm434 ura3::l imm434 (pACL-2)	This study	
RH29	ura3::l imm434 ura3::l imm434 (pACL-3)	This study	
pSM2	URA3 integrating plasmid	[6]	
pACL1	pTEF2, full-length CYR11373 in pSM2	This study	
pACL2	pTEF2, full-length CYR11334 in pSM2	This study	
pACL3	pTEF2, full-length CYR11377 in pSM2	This study	
pSMTC	pTEF2, full-length CYR1 in pSM2	This study	
NCE103 Blast	Nce103 HisG-ura3-HisG	[5]	


1. Gillum AM, Tsay EYH, Kirsch DR (1984) Isolation of the Candida albicans gene for orotidine-5′-phosphate decarboxylase by complementation of S. cerevisiae ura3 and E. coli pyrF mutations. Mol and Gen Genet 198: 179-182.
2. Fonzi WA, Irwin MY (1993) Isogenic Strain Construction and Gene Mapping in Candida albicans. Genetics 134: 717-728.
3. Davis DA, Bruno VM, Loza L, Filler SG, Mitchell AP (2002) Candida albicans Mds3p, a Conserved Regulator of pH Responses and Virulence Identified Through Insertional Mutagenesis. Genetics 162: 1573-1581.
4. Rocha CRC, Schroppel K, Harcus D, Marcil A, Dignard D, et al. (2001) Signaling through Adenylyl Cyclase Is Essential for Hyphal Growth and Virulence in the Pathogenic Fungus Candida albicans. Mol Biol Cell 12: 3631-3643.
5. Klengel T, Liang W-J, Chaloupka J, Ruoff C, Schroppel K, et al. (2005) Fungal Adenylyl Cyclase Integrates CO2 Sensing with cAMP Signaling and Virulence. Curr Biol 15: 2021-2026.
6. El Barkani A, Kurzai O, Fonzi WA, Ramon A, Porta A, et al. (2000) Dominant Active Alleles of RIM101 (PRR2) Bypass the pH Restriction on Filamentation of Candida albicans. Mol Cell Biol 20: 4635-4647.
